# Solid Matrix-Supported Supercritical CO_2_ Enhances Extraction of γ-Linolenic Acid from the Cyanobacterium *Arthrospira* (*Spirulina*) *platensis* and Bioactivity Evaluation of the Molecule in Zebrafish

**DOI:** 10.3390/md17040203

**Published:** 2019-03-30

**Authors:** Xiaohong Yang, Yi Li, Yanhua Li, Ding Ye, Li Yuan, Yonghua Sun, Danxiang Han, Qiang Hu

**Affiliations:** 1 Center for Microalgal Biotechnology and Biofuels, Institute of Hydrobiology, Chinese Academy of Sciences, Wuhan 430072, China; xhyang@ihb.ac.cn (X.Y.); yanhuali@ihb.ac.cn (Y.L.); liyuan@ihb.ac.cn (L.Y.); 2 State Key Laboratory of Freshwater Ecology and Biotechnology, Institute of Hydrobiology, Chinese Academy of Sciences, Wuhan 430072, China; liyi_0831@126.com (Y.L.); yeding@ihb.ac.cn (D.Y.); yhsun@ihb.ac.cn (Y.S.); 3 Laboratory for Marine Biology and Biotechnology, Qingdao National Laboratory for Marine Science and Technology, Qingdao 266237, China; 4 Key Laboratory for Algal Biology, Institute of Hydrobiology, Chinese Academy of Sciences, Wuhan 430072, China; 5 College of Life Sciences, University of Chinese Academy of Sciences, Beijing 100049, China; 6 College of Advanced Agricultural Sciences, University of Chinese Academy of Sciences, The Innovative Academy of Seed Design, Chinese Academy of Sciences, Beijing 100049, China; 7 Beijing Key Laboratory of Algae Biomass, Microalgae Biotechnology Center, SDIC Biotech Investment Co., LTD., State Development & Investment Corp., Beijing 100142, China

**Keywords:** *Arthrospira* (*Spirulina*) *platensis*, porous materials, diatomite, solid matrix-supported supercritical CO_2_ (SMSSC-CO_2_), γ-linolenic acid (GLA), bioactivity, zebrafish

## Abstract

Marine cyanobacteria represent a large untapped source of functional glycolipids enriched with polyunsaturated fatty acids (PUFAs) for human health. However, advanced methods for scalable isolation of diverse species containing high-purity PUFA-rich glycolipids will have to be developed and their possible pharmaceutical and nutraceutical functions identified. This paper introduces a novel solid matrix-supported supercritical CO_2_ extraction method for scalable isolation of the PUFA γ-linolenic acid (GLA)-enriched glycolipids from the cyanobacterium *Arthrospira* (*Spirulina*) *platensis*, which has been the most widely used among microalgae in the nutraceutical and pharmaceutical industries. Of various porous materials studied, diatomite was the best to facilitate extraction of GLA-rich glycolipids, resulting in an extraction efficiency of 98%. Gamma-linolenic acid made up 35% of total fatty acids (TFAs) in the extracts, which was considerably greater than that obtained with ethanol (26%), Bligh and Dyer (24%), and in situ transesterification (24%) methods, respectively. Lipidomics analysis revealed that GLA was exclusively associated with galactolipids. Pharmaceutical functions of GLA-rich galactolipids were investigated on a zebrafish caudal fin regeneration model. The results suggested that GLA extracted from *A. platensis* possessed anti-oxidative, anti-inflammatory, and anti-allergic activities, which acted in a concerted manner to promote post-injury regeneration of zebrafish.

## 1. Introduction

Gamma-linolenic acid (GLA) is an omega-6 polyunsaturated fatty acid (PUFA) that has shown multiple nutraceutical and pharmaceutical functions. Gamma-linolenic acid is a potential precursor in the biosynthesis of arachidonic acid (ARA), which possesses a wide range of biological activities related to its conversion to 20-carbon long-chain polyunsaturated fatty acid (C20 LC-PUFA), dihomo-gamma-linolenic acid (DGLA) [1]. Supplementing dietary GLA can increase serum DGLA, as well as serum ARA levels [2]. Also, GLA has been used to treat various medical conditions like rheumatoid arthritis [3,4], eczema [5], premenstrual syndrome [6,7], and reduction of nerve pain in people with diabetic neuropathy [8].

Many marine cyanobacteria contain considerable amounts of GLA in the form of glycolipids [9,10,11]. For example, the cyanobacterium *Arthrospira* (*Spirulina*) *platensis* can produce ca. 2% of GLA on a dry weight basis [12]. *Arthrospira platensis* has been commercially produced in large open raceway ponds using seawater [13,14] or alkaline water as a culture medium [15]. As GLA accounted for as high as 30% of total fatty acids (TFAs) in *A. platensis* cells [16], and α-linolenic acid (the isomer of GLA) was absent in TFAs [15], isolation, and purification of GLA-containing glycolipids from *A. platensis* could be cost-effective. In cyanobacteria glycolipids, 80 mol% are galactolipids [17], which contain the various fatty acid acyl groups (e.g., GLA) that are important for their functions. Although marine cyanobacteria represent an untapped natural source of glycolipids for pharmaceutical and nutraceutical applications, knowledge aboutcyanobacteria-derived glycolipids is rather limited. *Arthrospira platensis* has been subjected to the most extensive and intensive studies with regard to the health benefits of its biologically active compounds [14,18]. It may serve as an ideal model system to better understand the function of glycolipids in general and marine cyanobacteria in particular [19,20,21].

Extraction of lipids from *A. platensis* biomass is a crucial step toward isolation and purification of GLA [22,23,24]. Bligh and Dyer and Folch extraction methods are commonly applied to extraction of lipids from numerous microalgae including *A. platensis* under laboratory conditions [25]. Considering that GLA is produced for human consumption, the extraction processes require the use of solvents safe for human health and the environment. However, chloroform and methanol used in the conventional approaches can be toxic to both workers and consumers. Therefore, searching for more bio-safe and cost-effective solvents is imperative for extraction of bioproducts, particularly for human consumption.

Supercritical fluid extraction is an advanced technique that has recently been applied for extraction of biologically active products from microalgae under laboratory conditions [26,27,28,29,30]. The main supercritical solvent used is CO_2_, which is accessible, environmentally friendly, and generally recognized as safe by the U.S. Food and Drug Administration [31]. Carbon dioxide is easily removed from extractants and gives solvent-free end-products. Despite numerous advantages, supercritical CO_2_ (SC-CO_2_) has a main drawback, i.e., low polarity of CO_2_ that may lead to low extraction efficiency. Aiming at this challenge, a co-solvent ethanol was used as a polar modifier to alter the polarity of the supercritical fluid and enhance its solvating power towards targeted products. Previous studies addressed the effect of operational variables (e.g., temperature, pressure, CO_2_, and co-solvent consumption) on extraction efficiency of GLA [27,28,29,30,32,33,34], but the extraction efficiency remained relatively low (25–78%).

Previously, many nutraceutical studies of *A. platensis* were conducted with whole biomass or crude extracts of *A. platensis* [35,36]. To better explore the therapeutic potentials of *A. platensis*, functions of the refined bioproducts (e.g., GLA) from *A. platensis* biomass need to be demonstrated by employing an advanced toolbox. The functions of crude GLA extracted from *A. platensis* were studied using various cell line models [37,38]. To bridge the gap between the in vitro cellular level studies and nutraceutical or therapeutic applications, appropriate animal models and in vivo studies are required. Zebrafish have become an effective and popular model organism for drug screening, drug efficacy evaluation, and dissecting molecular mechanisms of drug action, as approximately 82% of human disease-related genes have at least one zebrafish orthologue [39]. The zebrafish larval caudal fin regeneration model is a well-established system for studying the roles of the immune system in organ injury and regeneration [40], and thus can be utilized as an in vivo model to investigate the bioactivities of compounds of interest as immunomodulators.

This work aimed at improving extraction efficiency of GLA from *A. platensis* by introducing a porous solid material to the supercritical fluid extraction (SFE) method. We hypothesized that prior to SFE treatment, microalgal biomass pre-mixed with the right type of porous solid materials could increase the surface area of algal biomass accessible to a solvent (CO_2_ alone or with ethanol), which facilitates penetration of the solvent into algal cells, and thus results in an enhanced extraction efficiency of GLA-enriched lipids. To test our hypothesis, we pre-mixed algal powders with several porous solid materials (i.e., molecular sieve, macroporous resin, and diatomite), individually, with different weight ratios. Total lipids, TFAs, and GLA extraction efficiencies were measured and compared with the control experiments that used algal powders only. Bioactivities of GLA obtained in this study were evaluated using a zebrafish caudal fin regeneration model.

## 2. Results and Discussion

### 2.1. Quantitative Analysis of Total Lipids and Fatty Acid Composition of A. platensis

Four methods were assessed for extraction of total lipids and GLA from *A. platensis*. They were (1) in situ transesterification followed by gas chromatography-mass spectrometry (GC-MS) analysis; (2) the Bligh and Dyer method; (3) extraction by ethanol with the aid of mechanical stirring; and (4) solid matrix-supported supercritical CO_2_ (SMSSC-CO_2_). The lipid content of *A. platensis* was 11.52 ± 0.11% of dry cellular weight (DCW), as determined by the Bligh and Dyer method. A slightly lower lipid content of 10.22 ± 0.17% DCW was obtained by extraction of algal biomass with ethanol. The SMSSC-CO_2_ method yielded the lowest amount of lipids (9.36 ± 0.30% DCW), but resulted in the greatest amounts of GLA on per TFAs and per total lipids basis (Table 1). In situ transesterification, followed by GC-MS analysis, showed that the TFAs content was 5.25 ± 0.07% DCW, and consisted of C16:0 (45.82% of TFAs), C16:1 (5.78%), C18:0 (0.64%), C18:1trans (1.41%), C18:1cis (0.22%), C18:2trans (0.15%), C18:2cis (21.99%), and C18:3n6 (GLA, 23.98%). Gamma-linolenic acid accounted for up to 1.26 ± 0.04% DCW and 1.18 ± 0.03% DCW for in situ transesterification and Bligh and Dyer methods, respectively (Table 1). Thus, the Bligh and Dyer method was selected to evaluate the GLA recovery in following studies.

### 2.2. Effect of Incorporated Solid Support on GLA Extraction Efficiency

**Figure 1:**
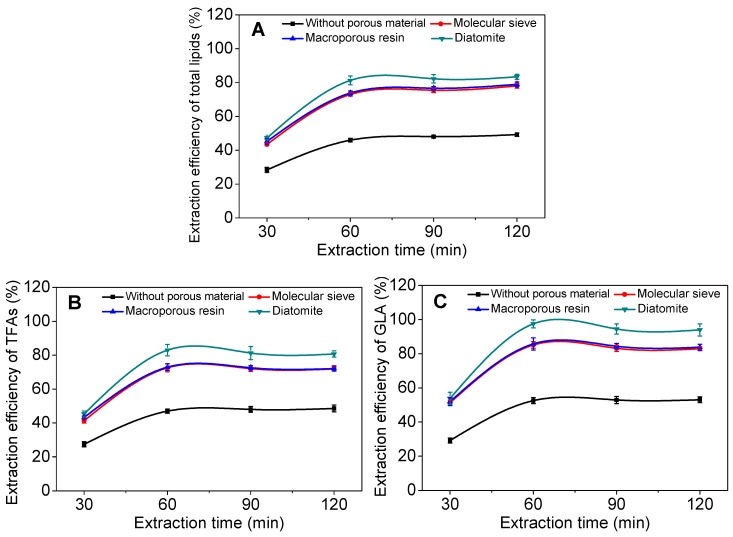
Effect of different solid supporting materials on extraction efficiencies of total lipids (**A**), total fatty acids (TFAs) (**B**), and γ-linolenic acid (GLA) (**C**) over extraction time. Experiments were performed under the following conditions: solid materials-to-algal biomass ratio, 1:2 (*w/w*); water content of solid materials, 60 wt.%; ethanol-to-biomass ratio, 3:1 (*v/w*); temperature, 40 °C; pressure, 41 MPa; CO_2_-to-mass ratio, 2.0 mL g^−1^ min^−1^. Error bars represent the standard deviation (*n* = 3).

Figure 1 shows that the introduction of a solid material (e.g., molecular sieve, macroporous resin, or diatomite) remarkably increased the extraction efficiencies of total lipids, TFAs, and GLA, when compared to the control without addition of such materials. It is believed that mixing algal powder with porous solids may increase bulk volume of algal biomass, therefore reducing the dead volume that exists between algal biomass and the internal boundary of the extraction vessel (particularly under supercritical state), thereby increasing the contact area between solvents and the algal-porous solid bed. Moreover, good dispersion of algal cells with porous solid materials may create a highly effective volume between algal cells and solvents, which is dependent on a desirable mixed-weight ratio of the porous solid material to algal biomass (Figure S1).

Among the three solid support materials tested, diatomite resulted in the highest extraction efficiencies of 81%, 83%, and 98%, for total lipids, TFAs, and GLA, respectively. These results were in large contrast to the control experiments (without porous materials) where the extraction efficiencies of total lipids, TFAs, and GLA were 46%, 47%, and 53%, respectively (Figure 1). Under the supercritical conditions, algal powder loaded inside the reaction vessel was compressed and clogged (mainly due to high pressure) to form a dense bed rather than a porous structure, which reduced the penetration (by diffusion and dispersion) of the solvents (CO_2_ and ethanol) into the cells, and thus decreased mass transfer of the solvents into individual algal cells. The improved extraction efficiencies that resulted from pre-mixing of algal biomass with solid porous materials may be contributable to increased porosity, and thus the surface-to-volume ratio of algal biomass by the porous materials, which in turn facilitated the solvents (liquid CO_2_ alone or with ethanol) penetrating into algal cells. The porous materials also enhanced the repartition of biomass matter leading to a more intimate contact between the supercritical solvents (CO_2_ + ethanol) and microalgal cells, leading to a higher extraction kinetics. Moreover, the recovery of GLA was much greater than that of total lipids and TFAs. The shape of the curves show that the porous materials play a role in the solubility of some compounds towards supercritical CO_2_ since the extract composition illustrates a better selectivity, particularly for GLA. That means GLA-rich lipid molecules were more readily extractable than non-GLA containing ones under the given conditions.

**Figure 2:**
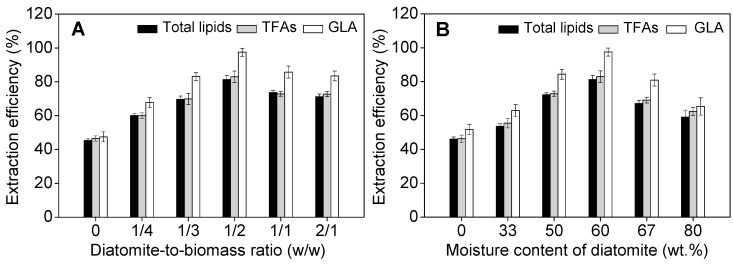
Effect of diatomite-to-biomass ratio (**A**) and moisture content of diatomite (**B**) on extraction efficiencies of total lipids, total fatty acids (TFAs), and γ-linolenic acid (GLA). Experiments were performed under the following conditions: ethanol-to-biomass ratio, 3:1 (*v/w*); temperature, 40 °C; pressure, 41 MPa; extraction time, 60 min (static extraction for 30 min + dynamic 30 min); CO_2_-to-mass ratio, 2.0 mL g^−1^ min^−1^. Error bars represent the standard deviation (*n* = 3).

As diatomite was superior to the other two porous materials, it was used for further study. Mixing of diatomite and biomass at a ratio of 1:2 (*w/w*) was the optimal for extraction of total lipids and GLA, resulting in the extraction efficiencies of 81% and 98% for total lipids and GLA, respectively. Smaller or greater (e.g., 0, 1:4, 1:3, 1:2, 1:1, and 2:1) than the optimal ratio resulted in reduced extraction efficiencies of total lipids and TFAs, as well as the recovery of GLA (Figure 2A). This may be attributable to increased dense biomass patches in the extraction vessel when the amount of diatomite was relatively too small, which prevents the penetration of solvents into algal cells. When porous solid materials were over-loaded, it resulted in the reduction of effective volume and contact area of algal cells and solvents.

Furthermore, the moisture content of diatomite was also found to significantly affect lipid extraction efficiency. The extraction efficiency of GLA increased from 52% to the maximum value of 98%, when the water content of diatomite increased from 0 to 60 wt.%. However, further increasing the water content to 80 wt.% resulted in decreased extraction efficiency to 65% (Figure 2B). It was speculated that soaking diatomite (containing 60 wt.% water) may have altered the polarity of the solvent mixture, i.e., CO_2_–ethanol–water, which facilitated the extraction of less-polar lipids galactolipids containing predominant amounts of GLA in the cells [29]. However, further increasing the water content to 80 wt.% might have brought about the formation of water film on the surface of the solid support and algal biomass, which reduced the mass transfer of ethanol and liquid CO_2_ into algal cells, and thus reduced overall lipid extraction. The extraction of GLA followed the similar trends as total lipids and TFAs over the investigated range of the water content of diatomite (Figure 2B).

When the SMSSC-CO_2_ approach was applied, the ratio of GLA in extracted TFAs increased to 35%, which was comparable with the record figure of 35.5% in the previous studies that employed direct transesterification on the freeze-dried material (Table 2). The recovery of GLA (based on the Bligh and Dyer method) by SMSSC-CO_2_ was virtually complete, which reached as high as 98%. Although glass wool [27,28], sea sand [30], and glass microspheres [32] were used to partially fill up the extraction vessel in order to eliminate the dead volume, and the recovery of lipids and GLA from these treatments was not examined in detail. On the other hand, these materials mixed into the *A. platensis* biomass will be introducing more problems, such that algal residues can be hardly separated from the dispersing agent after extraction. Compared with the microspheres and sea sand used in previous studies, diatomite offers an additional benefit of being safe for livestock. Diatomite represent a popular feed additive for livestock to ameliorate nutrient deficiency and illness problems [41]. Also, after lipid extraction, residues enriched with protein and carbohydrate can be applied to the animal feed industry [42,43].

### 2.3. Effect of Operational Parameters on GLA Extraction Efficiency

**Figure 3:**
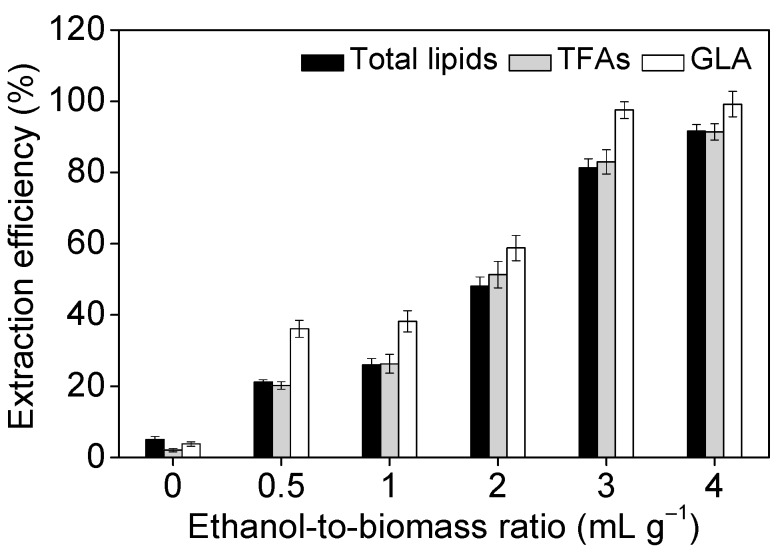
Effect of ethanol-to-biomass ratio on extraction efficiencies of total lipids, total fatty acids (TFAs), and γ-linolenic acid (GLA) in the presence of diatomite. Experiments were performed under the following conditions: diatomite-to-biomass ratio, 1:2 (*w/w*); water content of solid materials, 60 wt.%; temperature, 40 °C; pressure, 41 MPa; extraction time, 60 min (static extraction for 30 min + dynamic 30 min); CO_2_-to-mass ratio, 2 mL g^−1^ min^−1^. Error bars represent the standard deviation (*n* = 3).

As presented in Figure 3, there were almost no polar lipids recovered from the algal biomass after supercritical CO_2_ treatment in the absence of ethanol as the co-solvent, but the extraction efficiency of total lipids and GLA increased gradually from 5% and 4% to 92% and 99%, respectively, when ethanol was introduced to the biomass with a ratio of 3:1 (*v/w*). Our results were similar to those reported by Sajilata et al. [27] and Mendes et al. [29], in which introducing ethanol as a co-solvent increased the partitioning of GLA into the galactolipids fraction. Supercritical CO_2_ alone can extract 100% of neutral lipids contained in microalgae provided the biomass is previously dried and the extraction pressure is higher than 30 MPa. It is attributable to increasing the polarity of the CO_2_–ethanol mixture relative to CO_2_ only, thereby improving the extractability of algal biomass and accelerating partitioning of polar lipids (including GLA) into the solvent phase [44]. The extraction efficiencies of total lipids and TFAs at the ethanol-to-biomass ratio of 3:1 were 81% and 83%, respectively, and yet greater extraction efficiencies of total lipids and TFAs were 92% and 91%, respectively, when the ethanol-to-biomass ratio increase further to 4:1. As far as GLA was concerned, however, the extraction efficiency at the ethanol-to-biomass ratios of 3:1 and 4:1 was the same, i.e., 98 ± 1.2%. From an economic point of view, the ethanol-to-biomass ratio of 3:1 is sufficient to extract more than 97% of GLA from *A. platensis*.

Ethanol as an ideal solvent used to extract total lipids could provide a reasonable partition co-efficiency, forcing the solute to migrate to the solvent phase [45]. Principally, hydrophobic lipids (e.g., neutral lipids) are preferably partitioned into the non-polar solvent phase, whereas polar lipids are not so easy to dissolve in non-polar solvents due to their binding with the biomass matrix [46]. Therefore, co-solvents are applied to break the linkages between the polar lipids and biomass, leading to the increased solubility of the polar lipids. Both ethanol and liquid CO_2_ are generally recognized as safe solvents and easy to be removed from GLA, total lipids have a high partition coefficient in the ethanol.

**Figure 4:**
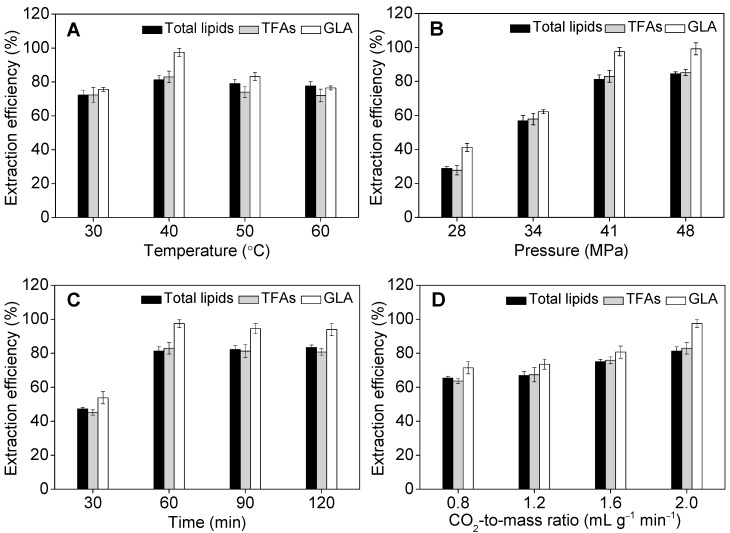
Effect of temperature (**A**), pressure (**B**), time (**C**), and CO_2_-to-mass ratio (**D**) on extraction efficiencies of total lipids, total fatty acids (TFAs), and γ-linolenic acid (GLA) by solid matrix-supported supercritical CO_2_ (SMSSC-CO_2_) extraction. Experiments were performed under the following conditions: diatomite-to-biomass ratio, 1:2 (*w/w*); water content of solid materials, 60 wt.%; ethanol-to-biomass ratio, 3:1 (*v/w*); and (**A**) pressure = 41 MPa, extraction time = 60 min (static extraction for 30 min + dynamic 30 min), CO_2_-to-mass ratio = 2.0 mL g^−1^ min^−1^; (**B**) temperature = 40 °C; extraction time = 60 min (static extraction for 30 min + dynamic 30 min), CO_2_-to-mass ratio = 2.0 mL g^−1^ min^−1^; (**C**) temperature = 40 °C, pressure = 41 MPa, CO_2_-to-mass ratio = 2.0 mL g^−1^ min^−1^; (**D**) temperature = 40 °C, pressure = 41 MPa, extraction time = 60 min (static extraction for 30 min + dynamic 30 min). Error bars represent the standard deviation (*n* = 3).

Figure 4A showed that the maximum recovery of GLA was obtained at the extraction temperature of 40 °C, which was remarkably higher than that at 30 °C. The extraction efficiency obtained at 30 °C was low because the solvent was not under supercritical conditions. However, further increasing temperatures to 50 °C and 60 °C resulted in a noticeable decrease in the efficiency. Depending on the pressure, the decrease of lipid yields from 40 °C up to 60 °C may be due to a retrograde solubility phenomenon. The optimal temperature identified in this study was the same as that reported by Sajilata et al. [27]. It is believed that a higher temperature than the optimum may increase the ethanol evaporation rate, which leads to the diffusion of ethanol from the liquid phase into the gas phase, resulting in reduced lipid extraction efficiency, in particular of GLA-containing molecules (Figure 4A). Moreover, the supercritical CO_2_ extraction method is operated at low temperature using a non-oxidant medium, hence it is particularly suitable for the extraction and separation of heat-sensitive and easily oxidized substances (particularly bioactive compounds) [47]. Data presented in Figure 4B showed that the extraction efficiency of GLA increased dramatically when the pressure increased from 28 MPa to 34 MPa, and yet greater extraction efficiencies of 98% and 99% were obtained at 41 MPa and 48 MPa, respectively. It is obvious that lipid yields are improved when pressure increases due to a higher density lead to a solubility increase. However, where energy consumption was concerned, the pressure was set at 41 MPa for further investigation. Note that the optimal pressure level determined in this study is similar to that applied for supercritical CO_2_-based extraction of astaxanthin from the green microalga *Haematococcus pluvialis* [48].

Lipid extraction efficiency is time-dependent, when the extraction duration increased from 30 min (15 min for main extraction and 15 min for draining) to 60 min (30 min for main extraction and 30 min for draining), the extraction efficiency of GLA increased from 54% to 98% (Figure 4C). Further extending extraction time from 90 min (30 min for main extraction and 60 min for draining) to 120 min (30 min for main extraction and 90 min for draining) resulted in gradual decrease of extraction kinetics, and the extraction efficiency of GLA reached a plateau. The same trend was observed for total lipids and TFAs (Figure 4C). The optimal extraction time of 60 min was shorter than that described in the literature, which ranges from 3 h to 9 h [49]. The extraction efficiency of GLA gradually increased from 71% to 98% when the consumption of CO_2_ increased from 0.8 to 2.0 mL g^−1^ min^−1^ (Figure 4D). It is likely due to enhanced elution of extracted lipids from biomass residues by passing greater amounts of the main solvent, i.e., liquid CO_2_ through both the extraction and collection vessels. Taken together, the key process parameters of extraction temperature, pressure, time, and CO_2_-to-mass ratio under investigation of GLA yields were established as 40 °C, 41 MPa, 60 min, and 2.0 mL g^−1^ min^−1^, respectively. SMSSC-CO_2_ at a temperature moderately above room temperature (40 °C), using CO_2_ as a green solvent, avoided the decomposition of heat-sensitive and easily oxidized substances to ensure the high quality and stability of products. Therefore, supercritical fluid extraction has more potential compared with other solvents extraction methods, as it is a fast and efficient unconventional extraction method developed for extraction of compounds of interest from solid matrices [50].

### 2.4. Distribution of GLA in Different Fractions

After supercritical extraction, total lipids were partitioned into three fractions, i.e., (1) lipid extracts obtained from the collection vessel; (2) lipids from the bottom of the kettle and the surface of sample basket eluted by ethanol, followed by filtering through a micro-pore bag; and (3) lipids remaining inside the residual biomass. As shown in Table 3, GLA was mainly present in the extracted lipids, accounting for 94.31% of the total GLA. The fraction eluted by ethanol from the bottom of the kettle and the surface of the sample basket accounted for 3.25% of the total GLA, whereas GLA in the remaining residual biomass was 2.44%, which is an inevitable loss and cannot be extracted using the current technique and process. Combining the lipid fractions 1 and 2 resulted in an extraction efficiency of 97.56% (94.31% + 3.25%), which was the highest reported thus far in the literature. Note that TFAs (fatty acid methyl esters equivalent) in the residual biomass was 19.35% (Table 3), which was mainly composed of fatty acids like C16:0, C16:1, and C18:0, suggesting that GLA-containing molecules were relatively more extractable than many other lipid molecules in *A. platensis* cells. Considering that biomass residues after lipid extraction may contain large amounts of proteins and carbohydrates along with valuable trace elements, the exploitation of algal biomass residues as animal feed additives or bio-fertilizers may deserve attention.

**Figure 5:**
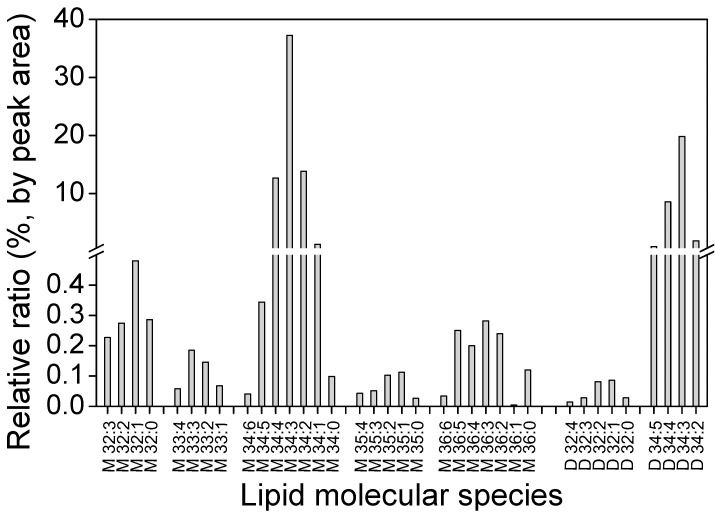
Relative ratio of MGDG and DGDG and molecular species profiles of MGDG and DGDG in lipid extracts of *A. platensis*. M, MGDG; D, DGDG.

Major cyanobacterial lipids are monogalactosyldiacylglycerol (MGDG), digalactosyldiacylglycerol (DGDG), sulfoquinovosyl diacylglycerol (SQDG), and phosphatidylglycerol (PG) [10]. The GLA-containing galactolipid extracts obtained from supercritical CO_2_ extraction of *A. platensis* were analyzed using liquid chromatography-tandem mass spectroscopy (LC-MS/MS) in the positive mode with an electrospray ionization (ESI) source. Electrospray ionization is a soft ionization technique that accomplishes the transfer of ions from solution to a gas phase [51]. Prior separation with liquid chromatography can facilitate detection of analytes in complex biological samples [52]. A total of 36 molecular species of galactolipids were identified by tandem MS, and the profiles and composition of each molecular lipid species of MGDG and DGDG in lipid extracts are shown in Figure 5. The dominant lipid molecules of *A. platensis* galactolipids and phospholipids. The bioactivity of polar lipids depends on the content and structure of fatty acryl groups, namely because ω-6 are essential for human health. Many glycolipids and phospholipids have been described to alleviate senescence, improve cognitive functions, and treat inflammatory diseases [53].

In this study, we uncovered that GLA was chiefly distributed in galactolipids in the form of a fatty acyl group (C18:3) among several classes of determined glycerolipids. According to the fragmentation patterns, 27 MGDG and 9 DGDG molecular species were identified in *A. platensis*. There are 17 species of galactolipids containing GLA (C18:3), which accounted for 47% of extracted lipids. It was revealed that the single most abundant fatty acyl chain in galactolipids is GLA (C18:3). Table 4 shows that GLA-containing MGDGs are mainly M34:4 (16:1–18:3) and M34:3 (16:0–18:3), making up 53% of total MGDG (Figure 5). The GLA-containing DGDG were mainly D34:5 (16:2–18:3), D34:4 (16:1–18:3), and D34:3 (16:0–18:3), which accounted for 29% of total DGDG. In addition, a trace amount of PG-containing GLA was detected, which was identified as PG 16:0–18:3. Otherwise, no other phospholipids contained GLA. The presence of the above bioactive fatty acyl groups demonstrated new types of lipids in *A. platensis* with respect to the configuration of lipids.

### 2.5. Recovery of GLA Using Different Extraction Methods

**Figure 6:**
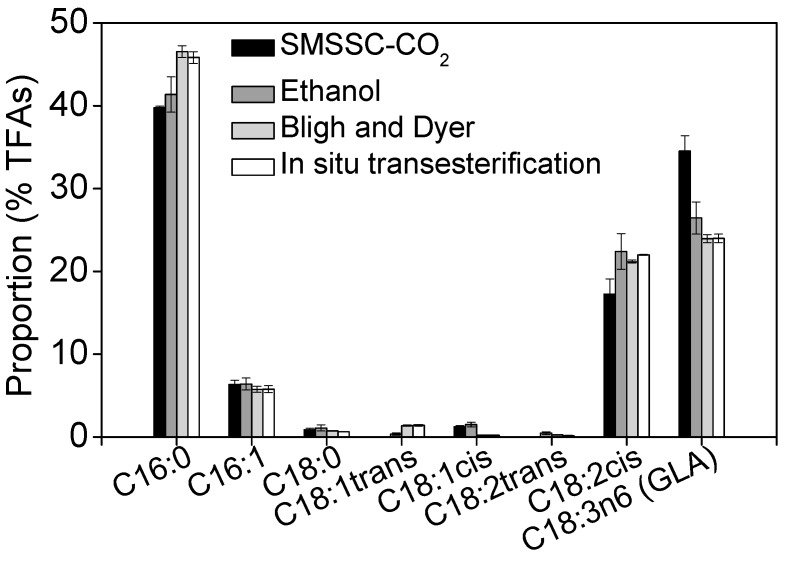
Fatty acid profiles obtained with different extraction methods. Error bars represent the standard deviation (*n* = 3).

In order to obtain lipid extracts with high concentrations of GLA, four extraction procedures were compared, i.e., SMSSC-CO_2_, ethanol (extraction with ethanol under mechanical stirring), Bligh and Dyer, and in situ transesterification method (Table 1 and Figure 6). Although the yields of total lipids and fatty acids obtained with the SMSSC-CO_2_ approach were lower by 23% and 38% than those obtained using Bligh and Dyer and in situ transesterification methods, respectively, the yield of GLA using the former method (SMSSC-CO_2_) was 1.16% DCW, which was comparable to that of the latter two methods (1.18% DCW and 1.26% DCW for Bligh and Dyer and in situ transesterification methods, respectively) (Table 1). Moreover, the data illustrated in Figure 6 revealed that the SMSSC-CO_2_ resulted in remarkably greater content of GLA (34.52%) as compared to that (23.95–26.44%) obtained by the other three approaches, and was 67% greater than that reported by Gunstone [54]. It was demonstrated that SMSSC-CO_2_ had greater extraction selectivity for GLA than the other three methods tested.

On the other hand, ethanol, Bligh and Dyer, and in situ transesterification methods were found to exert greater extraction selectivity for palmitic acid (C16:0) and linoleic acid (C18:2cis) (Figure 6 and Figure S2). A possible reason is that the porous solid materials used in SMSSC-CO_2_ might have served as an absorbent to absorb impurities such as saturated fatty acids and pigments, making the downstream purification of crude GLA-containing lipids easier incidentally. This finding indicated that SMSSC-CO_2_ is a promising technology particularly for GLA extraction from *A. platensis* biomass, due to its greater extraction selectivity for GLA, and its use of green solvents including CO_2_ and ethanol. It is considered more environmentally friendly compared to the Bligh and Dyer method that involves harmful solvents including chloroform and methanol. The technology developed in this study can not only guide for designing a large-scale GLA extraction process, but also be applicable for extraction of low-polarity lipophilic compounds, like polyphenols, polysaccharides, carotenoids, and polyunsaturated fatty acids from marine microalgae or higher plants [55].

### 2.6. Bioactivity Test of GLA Using a Zebrafish Caudal Fin Regeneration Model

**Figure 7:**
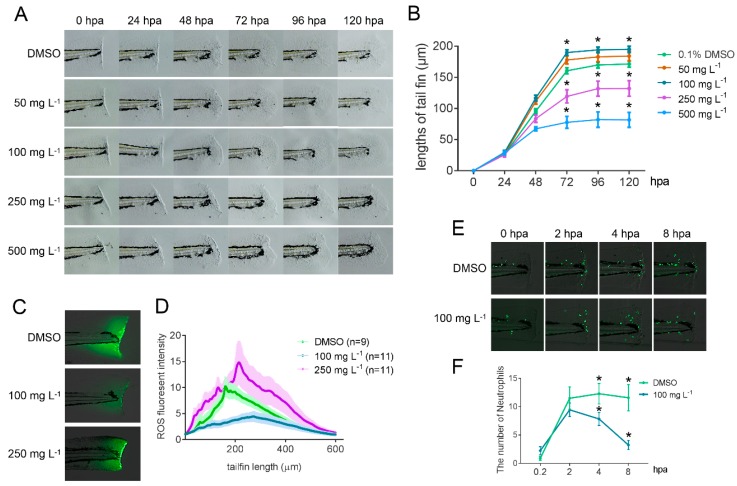
Bioactivities of GLA tested on a zebrafish caudal fin regeneration model. (**A–B**) Effects of 50, 100, 250, and 500 mg L^−1^ of GLA on zebrafish caudal fin regeneration. Proper concentrations (50 and 100 mg L^−1^) of GLA promoted the regeneration of early fin primordia of zebrafish larvae, whereas higher concentrations (250 and 500 mg L^−1^) did not. (**C–D**) Reactive oxygen species (ROS) test of wounded zebrafish treated with DMSO, 100 and 250 mg L^−1^ of GLA. Proper concentration (100 mg L^−1^) of GLA reduced oxidative stress at the wound region, whereas higher concentration (250 mg L^−1^) did not. (**E–F**) Neutrophil attraction of wounded zebrafish caudal fin treated with DMSO and 100 mg L^−1^ of GLA. Proper concentration (100 mg L^−1^) of GLA reduced the number of neutrophils at the wound region revealing the anti-inflammatory effect of this fatty acid. Asterisks (*) indicate statistically significant differences compared with the DMSO (*t*-test, *p* < 0.05).

Gamma-linolenic acid extracted from *A. platensis* biomass was used for bioactivity test in a zebrafish caudal fin regeneration assay. The caudal fin of wild-type AB zebrafish at 54-h post fertilization (hpf) were amputated, and the amputated zebrafish were raised individually in the different concentrations (i.e., 50, 100, 250, 500 mg L^−1^) of GLA extracts. Compared to the dimethyl sulfoxide (DMSO) control, the addition of 50 and 100 mg L^−1^ of GLA accelerated the regeneration of the wound tissue obviously, but a higher concentration than 250 mg L^−1^ of GLA decelerated the regeneration (Figure 7A,B). In order to reveal the mechanism underlying this phenomenon, a reactive oxygen species (ROS) assay and a neutrophil attraction assay were performed. The results showed that 100 mg L^−1^ of GLA exhibited strong oxygen free radical scavenging activity, but 250 mg L^−1^ of GLA did not (Figure 7C,D). Furthermore, both recruitment at the very beginning after amputation and subsequent clearance of neutrophil cells were more efficient in the zebrafish cultured in 100 mg L^−1^ of GLA when compared with the controls (Figure 7E,F). These results indicate that a proper concentration of GLA improved anti-oxidative capability of zebrafish. Meanwhile, it could also modulate the immune system of zebrafish to better respond to the external injury in a more efficient manner. All these results suggest that GLA has multiple-bioactivities, including anti-oxidation, anti-inflammation, and anti-allergy, which led to the accelerated regeneration of the wound in zebrafish.

Gamma-linolenic acid has been demonstrated to be effective in treating various disorders such as rheumatoid arthritis [3], diabetes, multiple trauma, and premenstrual syndrome [7]. Several studies have shown that different esterified forms of polyunsaturated fatty acids exhibited different levels of bioavailability and effectiveness of their bioactivities. For example, docosahexaenoic acid-phosphatidylcholine (DHA-PC) produced a more significant improvement in brain development, cognitive performance and emotional well-being than docosahexaenoic acid-triglyceride (DHA-TG) [56,57,58]. Moreover, DHA-PC exhibited better efficacy than the recombination of DHA-TG with egg PC or α-glycerylphosphorylcholine in various mice tissues [59]. We suspect that *A. platensis*-derived GLA, which is mostly present in the galactolipid molecules, may combine or surpass the bioactivities of galactolipids without GLA or GLA alone.

## 3. Materials and Methods

### 3.1. Materials and Reagents

*Arthrospira platensis* powders (5–6% moisture) were obtained from C.B.N. Spirulina Bio-engineering Inc. (Dongtai, Jiangsu Province, China). The standards of 37 fatty acid methyl esters (37 FAMEs, C4–C24, Sigma–Aldrich #18919-1AMP), tridecanoic acid (C_13_) standard (Sigma–Aldrich #91988-5G), pentadecane (C_15_) (Sigma–Aldrich #76509-5ML) were purchased from Sigma Chemical Co. (St Louis, MO, USA). Monogalactosyldiacylglycerol (MGDG), digalactosyldiacylglycerol (DGDG), and sulfoquinovosyl diacylglycerol (SQDG) were purchased from Avanti Polar Lipids, Inc. (Alabaster, USA). The physicochemical properties of molecular sieve (Nanda Synthetic, Jiangyin, China), HP 20 macroporous resin (SEPDEX, Zhengzhou, China), and diatomite (Thermo Scientific^TM^, Waltham, MA, USA) are listed in Table 5. Thin-layer chromatography silica gel 60 was purchased from Merck KGaA (Darmstadt, Germany). All extraction reagents were of analytical grade and the other reagents were of HPLC grade. Hydrochloric acid (36–38%) was purchased from Sinopharm Chemical Reagent Co. (Beijing, China). Wild-type of the AB strain and *Tg(mpx:EGFP)* transgenic zebrafish (Cat. ID: CZ13) [60] were purchased, maintained, and raised at the China Zebrafish Resource Center (CZRC, http://zfish.cn), Wuhan, China.

### 3.2. Methods

#### 3.2.1. Lipid Extraction

Total lipids of *A. platensis* powders were extracted and determined using a modified Bligh and Dyer method that was described in Laurens et al. [61]. Briefly, 100 mg of *A. platensis* powder was manually ground into fine powder in liquid nitrogen and transferred to a labeled glass vial. Ten milliliters of chloroform and methanol (2:1, *v/v*) mixture as extraction solvent was added into the vial and the mixture was kept stirring at 130 rpm for 1 h in a thermal incubator at a constant temperature of 30 °C. Then, 2.5 mL of 0.7% (*w/v*) potassium chloride was added into the vial and vortexed for 30 s, followed by centrifugation at 1000 *g* for 5 min at 20 °C to remove crude proteins. The chloroform phase containing crude lipids was manually collected and transferred into a pre-weighed vial. The extraction procedure was repeated once with addition of 4 mL chloroform and methanol (2:1, *v/v*) to the aliquot and vortexed for 30 s at room temperature. The chloroform phases from the two extractions were combined into a pre-weighed vial. The solvents in the vial were evaporated with the aid of nitrogen gas (NDK200-2, Lang Yue Instrument Manufacturing Co., Ltd., Changzhou, China) and remaining lipid extracts were lyophilized (LABCONCO^®^, Kansas City, USA) overnight. Lipids in the vial were weighed and total lipids were gravimetrically calculated on the basis of dry algal biomass. The extracted lipids were stored under −20 °C for fatty acids analysis. Error bars represent the standard deviation (*n* = 3).

#### 3.2.2. Determination of Fatty Acid Methyl Esters (FAMEs)

Fatty acid profiles in the algal biomass were determined by the protocol described by Van Wychen et al. [62] with slight modifications. In brief, 10 mg of algal biomass sample was added into a glass vial (Agilent Technologies, CA, USA) containing 300 µL hydrochloric acid and methanol mixtures (5%, *v/v*), 200 µL chloroform and methanol (2:1, *v/v*) solvents in the presence of 25 µL of tridecanoic acid (10 mg mL^−1^). Tridecanoic acid, an odd-chain fatty acid that does not naturally occur in algae, is transesterified with the sample to quantify the total FAMEs content on GC and thus can be used as a surrogate/recovery standard. The transesterification reaction took place at 85 °C for 1 h. The resulting FAMEs were extracted with 1 mL hexane (in the presence of 5 µL pentadecane as an internal standard) and analyzed by GC-MS for determination of fatty acid composition and content (further details are described in Section 3.3.1). Error bars represent the standard deviation (*n* = 3).

#### 3.2.3. Lipids and GLA Extraction with Ethanol under Mechanical Stirring

Ten grams of dried biomass were extracted with ethanol (liquid-to-solid ratio of 1:10, *w/v*) at 50 °C for 2 h under stirring in a round glass bottle. The mixture was centrifuged and supernatant was manually collected, followed by evaporation using a vacuum rotary evaporator (Rotavapor R-300, BÜCHI Labortechnik AG, Postfach, Switzerland) at a pressure of 0.015 MPa and a temperature of 45 °C. Total lipids were obtained by lyophilization (LABCONCO^®^, Kansas City, U.S.). The profile and content of fatty acids were analyzed with GC-MS (as described in Section 3.2.2 and Section 3.3.1). The extraction was repeated twice.

#### 3.2.4. Supercritical Fluid Extraction

**Figure 8:**
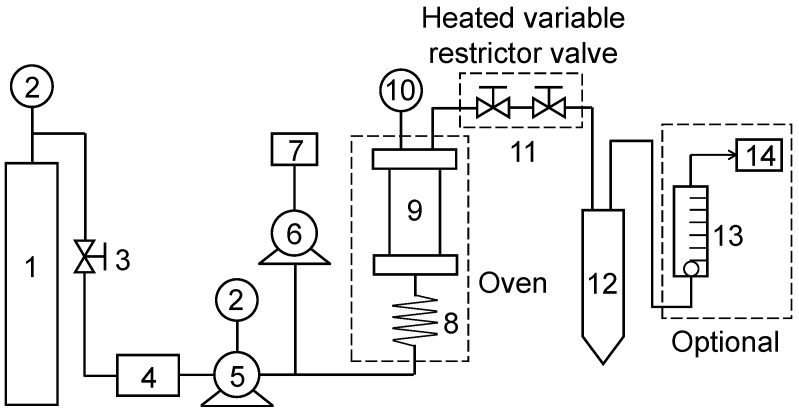
Single vessel configuration of the supercritical CO_2_ extraction apparatus: 1, liquid CO_2_ cylinder; 2, manometers; 3, check valve; 4, CO_2_ cooler; 5, 6, high-pressure pumps for pumping CO_2_ and co-solvent; 7, co-solvent addition; 8, preheat coil; 9, extraction vessel; 10, thermometer; 11, static/dynamic valve; 12, collection vial; 13, flow meter gas CO_2_; 14, CO_2_ vent.

Supercritical carbon dioxide (SC-CO_2_) extraction of lipids and GLA from *A. platensis* biomass was conducted in an SFT-110XW SFE apparatus (Supercritical Fluid Technologies, Inc., Delaware, USA) (Figure 8) using carbon dioxide (purity 99.99%) (Chengweifeng Technologies Inc., Beijing, China) as solvent. For each experiment, 10 g of spray-dried biomass with a particle size of 0.2 mm was subjected to a sample basket and loaded into a 100 mL extraction vessel. Briefly, liquid CO_2_ supplied from a gas cylinder (1) was cooled by ethanol (CO_2_-to-ethanol ratio = 20:1, *v/v*) to −5 °C before being pressurized to the desired pressure and passed through an extraction vessel (9), which contained dry *A. platensis* powder and support materials (solid form) mixed at different ratios. Extracted lipids were regularly collected at determined time intervals into a collection vessel (12), and were cooled to room temperature after the extractor was depressurized to the atmospheric pressure. Crude lipids bound onto the internal surface of the extraction vessel and residual biomass were recovered by washing with ethanol, and then mixed with the above extracted lipids. Total lipids were obtained by evaporation of solvent using a rotary evaporator and were quantified gravimetrically. Composition of fatty acids was analyzed using GC-MS (see Section 3.2.2 and Section 3.3.1).

To optimize the operation conditions for GLA yield, the lipids were extracted with variables that could affect GLA extraction efficiency, including solid support types (molecular sieve, macroporous resin, diatomite) (Table 5), solid-to-biomass ratio (0, 1:4, 1:3, 1:2, 1:1 and 2:1 *w/w*), moisture content of solid support (0, 33, 50, 60, 67 and 80 wt.%), ethanol-to-biomass ratio (0, 0.5:1, 1:1, 2:1, 3:1, and 4:1 *v/w*), extraction temperature (30, 40, 50, and 60 °C), extraction pressure (28, 34, 41, and 48 MPa), extraction time range from 30 min (15 min for main extraction and 15 min for draining), 60 min (30 min for main extraction and 30 min for draining), 90 min (30 min for main extraction and 60 min for draining), and 120 min (30 min for main extraction and 90 min for draining), and CO_2_-to-mass ratio (0.8, 1.2, 1.6, and 2.0 mL g^−1^ min^−1^).

#### 3.2.5. Determination of Yields of Total Lipids and GLA

Total lipids yield (Yield_Total lipids_), GLA yield (Yield_GLA_), and extraction efficiencies (or recoveries) of total lipids, TFAs, and GLA were calculated by using the following equations:

(1) $${\mathrm{Yield}}_{\mathrm{Total}\;\mathrm{lipids}}\;(\%\;\mathrm{DCW})=\frac{m_{\mathrm{Total}\;\mathrm{lipids}\;}\left(g\right)}{m_{\mathrm{Biomass}\;(\mathrm{DCW})\;}\left(g\right)}\times 100$$

(2) $${\mathrm{Yield}}_{\;\mathrm{GLA}}(\%\;\mathrm{DCW})=\frac{m_{\mathrm{GLA}\;}(g)}{m_{\mathrm{Biomass}\;(\mathrm{DCW})\;}(g)}\times 100$$

(3) $$\mathrm{Extraction}\;\mathrm{efficiency}\;\mathrm{or}\;\mathrm{recovery}\;(\%)=\frac{m_{{\mathrm{Total}\;\mathrm{lipids},\;\mathrm{TFAs}\;\mathrm{or}\;\mathrm{GLA}\;\mathrm{measured}\;\mathrm{via}\;\mathrm{SC}-\mathrm{CO}}_{2\;}(g)}}{m_{\mathrm{Total}\;\mathrm{lipids},\;\mathrm{TFAs}\;\mathrm{or}\;\mathrm{GLA}\;\mathrm{measured}\;\mathrm{via}\;\mathrm{Bligh}\;\mathrm{and}\;\mathrm{Dyer}\;\mathrm{method}\;(g)}}\times 100$$

#### 3.2.6. Zebrafish Caudal Fin Regeneration Assay

Wild-type of AB strain and transgenic zebrafish were purchased from, maintained and raised at the China Zebrafish Resource Center (CZRC, http://zfish.cn),Wuhan, China. Briefly, zebrafish were maintained at 28.5 °C with a 14/10 photoperiod of light/dark cycle and fed brine shrimp three times a day. All procedures relating to the care and use of fish were approved by the ethics committee from the Institute of Hydrobiology, Chinese Academy of Sciences on 28 May 2018 (Approval Protocol No. IHB20180528). The investigation conforms to the Guide for the Care and Use of Laboratory Animals published by the US National Institutes of Health.

The caudal fins of wild-type AB zebrafish were amputated at 54 hpf with a scalpel under a stereomicroscope (Olympus). The cutting site was at the tip of the caudal notochord along the vertical axis of the length. Amputated larval fish were raised individually in a 24-well plate with 2 mL different concentrations of GLA solution as indicated in the Results section and Figure 7A. Gamma-linolenic acid were diluted in DMSO (50 mg mL^−1^) as stock solution. The stock was further diluted with 0.3 × Danieau buffer, which 17.4 mM NaCl, 0.21 mM KCl, 0.12 mM MgSO_4_, 0.18 mM Ca(NO_3_)_2_, 1.5 mM 4-(2-hydroxyethyl)-1-piperazineethanesulfonic acid buffer (HEPES, pH 7.6), to make the working solution. Twelve larvae were treated with one concentration of working solution. For taking pictures, each larva was first anesthetized with 0.015% Tricaine (Sigma Cat. No: A-5040) in 0.3 × Danieau buffer and then mounted with 2% methylcellulose. The image was taken under a stereomicroscope (AF205, Leica). Images of every larva were taken at 0-, 12-, 24-, 48-, 72-, and 120-h post amputation (hpa). Embryos were recovered in 0.3 × Danieau buffer and put back into freshly prepared GLA solution after imaging.

For detection of ROS, 48-hpf wild-type larval fish were cultured in 2 mL of 0.1% DMSO, 50, 100, or 250 mg L^−1^ GLA in a 6-well plate with 10 larvae per well for 24 h. Then, the treated larvae were anesthetized with 0.015% Tricaine and their tails were wounded as previously described [40]. After tail injury, 10 larvae of one group were incubated in 1 mL of working solution of the ROS detection kit in the dark for 10 min (ENZO, Cat. No: ENZ-51042-K500), washed 3 times with HBSS, and then mounted with 2% methylcellulose for imaging. All images were taken under a Leica DMI 6000 microscope using a 10×/numerical aperture 0.3 objective with the same parameters. For measurement of fluorescence intensity of ROS, 8-bit images were imported into Fiji software and converted into 32-bit [63]. A rectangle tool was used to select the wound region and applied to the “ROI” manager which can be reused for other images. The function of the “Plot Profile” was used to measure the intensity of the selected region.

The *Tg(mpx:EGFP)* transgenic zebrafish was used in the live image analysis of neutrophil attraction assay. Forty-eight hpf larval fish were cultured in 10 mL of 0.1% DMSO or 100 mg L^−1^ GLA in a 6-well plate with 10 larvae per well for 24 h. Then, the treated larvae were anesthetized with 0.015% Tricaine and their tails were wounded as previously described [40]. After tail injury, the larval fish were embedded in 1% low-melting agarose and covered with 10 mL 0.3 × Danieau buffer in a 60-mm Petri dish at 28.5 °C. Images of each larva were taken at 20 min, 2, 4, and 8 h after tail injury under a Leica DMI-6000 microscope using a 10×/NA 0.3 objective. Images were imported into Fiji software [63] and the neutrophils at the wound site were counted.

### 3.3. Analytical Methods

#### 3.3.1. Fatty Acid Analysis

An Agilent 7890B+5977A GC/MS (Agilent Technologies Inc., USA) was used to determine the composition of FAMEs. The capillary column was HP-88 (60 m × 0.25 mm × 0.2 µm). Initial temperature of the oven was 50 °C and maintained for 2 min, heated at a rate of 25 °C min^−1^ to 175 °C, and maintained for 5 min, then heated again at a rate of 7 °C min^−1^ to 210 °C and maintained for 2 min, and finally heated at a rate of 2 °C min^−1^ to 230 °C and maintained for 1 min. The injector temperature was kept at 250 °C in split (20:1) mode for an injection volume of 1 µL. The auxiliary heater, electron ionization (EI) source, and MS Quadrupole temperature were 250 °C, 230 °C, and 150 °C, respectively. Helium was used as the carrying gas at 1 mL min^−1^.

#### 3.3.2. Polar Lipids Analysis

Qualitative analysis of galactolipids (including MGDG and DGDG) and phospholipids was performed with a Waters TQ-S triple quadruple electrospray ionization mass spectrometer (ESI-MS/MS) equipped with a Waters ACQUITY ultra-performance liquid chromatography (Waters, USA). Lipid extracts (30 mg) were dissolved in 2 mL chloroform and methanol (1:1, *v/v*) solutions, then filtered with a 0.22-μm nylon membrane to remove insoluble debris. The samples were diluted by 1000-fold progressively chloroform and methanol (1:1, *v/v*) before instrumental analysis [64,65].

## 4. Conclusions

A porous solid material supported supercritical-CO_2_ method was successfully developed for extraction of GLA-enriched glycolipids from the biotechnologically important cyanobacterium *A. platensis*. Under the optimal conditions, which included the solvent-to-solid ratio of 3:1 (*v/w*), diatomite-to-biomass ratio of 1:2 (*w/w*), CO_2_-to-mass ratio of 2.0 mL g^−1^ min^−1^, temperature of 40 °C, pressure of 41 MPa, and time of 60 min, the extraction efficiency of GLA can reach 98%, corresponding to 1.16% DCW. Gamma-linolenic acid made up 34.52% of TFAs, which was significantly greater than 26.44%, 23.95%, and 23.98% obtained with ethanol extraction in aid of mechanical stirring, Bligh and Dyer, and in situ transesterification methods, respectively. Moreover, SMSSC-CO_2_ using CO_2_ as a solvent and ethanol as a co-solvent at a low temperature (e.g., 40 °C) may better preserve heat-sensitive bioactive compounds in the cells, while at the same time be safer for production operators and the environment. Lipidomics analysis revealed that GLA was exclusively associated with galactolipids in *A. platensis*. Furthermore, we utilized a zebrafish model to demonstrate the bioactivities of *A. platensis* GLA as an anti-oxidant and immune system regulator. These findings highlight the application potentials of these new forms of GLA in nutraceutical and pharmaceutical industries.

## Figures and Tables

**Table 1:** Comparative analysis of total lipids, total fatty acids (TFAs), and γ-linolenic acid (GLA) from *Arthrospira platensis* by four extraction methods (means ± standard deviation).

Methods	Total Lipids Yield (% DCW)	Total Lipids Recovery (%)	GLA Yield (% DCW)	GLA Recovery (%)	GLA (% Total Lipids)	TFAs Yield (% DCW)	GLA (% TFAs)
In situ T	NA	NA	1.26 ± 0.04	100	NA	5.25 ± 0.07	23.98 ± 0.52
B & D	11.52 ± 0.11	100	1.18 ±0.03	100	10.33	4.97 ± 0.11	23.95 ± 0.47
Ethanol	10.22 ± 0.17	88.72	1.13 ± 0.06	94.96 ^a^	11.06	4.27 ± 0.16	26.44 ± 1.94
SMSSC-CO_2_	9.36 ± 0.30	81.25	1.16 ± 0.07	97.58 ^a^	12.39	3.80 ± 0.16	34.52 ± 1.88

In situ T: in situ transesterification; B & D: Bligh and Dyer method; Ethanol: extraction with ethanol; SMSSC-CO_2_: solid matrix-supported supercritical CO_2_; NA: not available; ^a^ Based on the data of the Bligh and Dyer method.

**Table 2:** Supercritical CO_2_ extraction of total lipids and GLA contents of several *Arthrospira* (*Spirulina*) species and strains.

Strains	Biomass Forms	Solvent	Pressure (MPa)	Temp. (°C)	Time (min)	CO_2_-to-Mass Ratio	Total Lipids (% DCW)	GLA (% DCW)	GLA (% TFAs)	GLA Recovery of B & D (or in situ T) (%)	Reference(s)
*Arthrospira platensis*	10 g spray-dried powder (0.2 mm) (with porous materials)	CO_2_ + ethanol	41	40	60	2 mL g^−1^ min^−1^	9.4	1.16	34.5	98 (92)	This study
*Spirulina platensis*	16 g freeze-dried (with glass wool), crushing (0.25 mm)	CO_2_ + 13.7 mL ethanol	40	40	60	700 mL min^−1^	8.6	0.64	15.8	100 (78)	[27]
*Arthrospira maxima*	5 g freeze-dried (with glass wool), crushing (0.2 mm)	CO_2_ + 10 mol% ethanol	35	60	NA	2 g min^−1^	NA	0.44	35.5	45	[28,29]
*Arthrospira platensis*	2 g biomass (with 4 g sea sand)	50% CO_2_ + ethanol	30	40	90	NA	6.7	0.90	6.5–11.7	25	[30]
*Spirulina platensis*	80 g freeze-dried (with 100 g glass microspheres), crushing (≤0.37 mm)	CO_2_	70	55	90	10 kg h^−1^	7.8	0.67	18.5–21.1	NA	[32]
*Spirulina platensis*	Dried powder	CO_2_	40	40	240	24 kg h^−1^	7.2	0.29	23.2	NA	[33]
*Arthrospira platensis*	Dried powder	CO_2_	30	60	NA	3 mL min^−1^	NA	0.04	NA	NA	[34]

B & D: Bligh and Dyer method; In situ T: in situ transesterification; NA: not available.

**Table 3:** Total lipids and GLA recovery estimated from different distribution compositions after SMSSC-CO_2_ extraction in duplicate (means ± standard deviation).

Samples	Total Lipids Yield (% DCW)	TFAs Yield (% DCW)	Percentage (%)	GLA Yield (% DCW)	Percentage (%)
Extraction vessel ^a^	9.36 ± 0.30	3.80 ± 0.16	72.80	1.16 ± 0.07	94.31
Washing ^b^	1.10 ± 0.04	0.41 ± 0.01	7.85	0.04 ± 0.00	3.25
Biomass residues ^c^	ND	1.01 ± 0.02	19.35	0.03 ± 0.00	2.44
Total	10.46	5.22	100	1.23	100

^a^ Collected in the collection vial; ^b^ Washing bottom of kettle and surface of sample basket with ethanol; ^c^ Lipids remaining in residual biomass were extracted via in situ transesterification method; ND: not determined.

**Table 4:** The molecular species of monogalactosyldiacylglycerol (MGDG) and digalactosyldiacylglycerol (DGDG) in *A. platensis* lipid extracts.

Lipids	MGDG Acyl Chain	Lipids	MGDG Acyl Chain
32:4	16:3–16:1; 16:4–16:0; 14:1–18:3; 14:2–18:2	33:5	16:2–17:3
32:3	16:3–16:0; 16:2–16:1; 14:0–18:3	33:4	16:1–17:3; 16:0–17:4
32:2	16:2–16:0; 16:1–16:1; 14:0–18:2	33:3	16:0–17:3
32:1	16:1–16:0	33:2	16:0–17:2
32:0	16:0–16:0	33:1	16:0–17:1
34:5	16:2–18:3; 16:1–18:4	35:5	16:1–19:4; 17:2–18:3
34:4	16:1–18:3	35:4	16:1–19:3; 17:1–18:3
34:3	16:0–18:3	35:3	16:1–19:2; 16:0–19:3
34:2	16:0–18:2	35:2	16:0–19:2; 16:1–19:1
34:1	16:0–18:1	35:1	16:0–19:1
34:0	16:0–18:0	35:0	16:0–19:0
36:6	18:3–18:3	36:2	16:0–20:2; 16:1–20:1; 18:2–18:0; 18:1–18:1
36:5	18:2–18:3	36:1	16:0–20:1; 16:1–20:0; 18:1–18:0
36:4	18:3–18:1; 18:2–18:2; 16:0–20:4; 16:1–20:3	36:0	16:0–20:0
36:3	16:0–20:3; 16:1–20:2; 18:3–18:0; 18:2–18:1		
**Lipids**	**DGDG acyl chain**	**Lipids**	**DGDG acyl chain**
34:5	16:2–18:3	32:3	NA
34:4	16:1–18:3	32:2	14:2–18:0; 15:2–17:0; 13:2–19:0
34:3	16:0–18:3	32:1	14:1–18:0; 13:1–19:0
34:2	16:0–18:2	32:0	NA

NA: not available.

**Table 5:** Physicochemical properties of porous materials.

Materials	Molecular Sieve ^a^	Macroporous Resin (HP 20) ^a^	Diatomite ^a^
Chemical composition	3/4CaO·1/4Na_2_O·Al_2_O_3_· 2SiO_2_·9/2H_2_O	Polystyrene benzene	SiO_2_ (>85%)
Particle size (µm)	2.5	420–840 (20–40 mesh)	590 (30 mesh)
Unit surface area (m^2^ g^−1^)	300–1000	500−600	40–65
Pore size (nm)	2–50	46	NA
Moisture content (%)	<2	45–60	0.11
Density (g cm^−3^)	0.64	1.05–1.09	0.47
Functional groups	OH^−^	OH^−^/H^+^	OH^−^
Hydrophobic/hydrophilic	Hydrophilic	Hydrophilic	Hydrophilic

^a^ Data were obtained from manufacturers; NA: not available.

## Supplementary Materials

The following are available online at https://www.mdpi.com/1660-3397/17/4/203/s1, Figure S1: Comparison of the states of mixing porous materials with algal powders and the microscopic images of residues (Scale bar 50 μm), Figure S2: The chromatogram of fatty acids obtained from four extraction methods.
