# Supplementary material for: Solid Matrix-Supported Supercritical CO2 Enhances Extraction of γ-Linolenic Acid from the Cyanobacterium Arthrospira (Spirulina) platensis and Bioactivity Evaluation of the Molecule in Zebrafish
Source: Mar Drugs. 2019 Mar 30;17(4):203. doi: 10.3390/md17040203 (PMC6520994; doi:10.3390/md17040203)
Supplement: Supplementary file 1 [file marinedrugs-17-00203-s001.pdf]

marine drugs

Article

Solid Matrix-Supported Supercritical CO_2 Enhances Extraction of γ-Linolenic Acid from the Cyanobacterium *Arthrospira* (*Spirulina*) *platensis* and Bioactivity Evaluation of the Molecule in Zebrafish

Xiaohong Yang [1,5], Yi Li [2,6], Yanhua Li [1], Ding Ye [2,6], Li Yuan [1], Yonghua Sun [2,6], Danxiang Han [1,4,*] and Qiang Hu [1,2,3,4,7,*]

1 Center for Microalgal Biotechnology and Biofuels, Institute of Hydrobiology, Chinese Academy of Sciences, Wuhan 430072, China; xhyang@ihb.ac.cn (X.Y.); yanhuali@ihb.ac.cn (Y.L.); liyuan@ihb.ac.cn (L.Y.)
2 State Key Laboratory of Freshwater Ecology and Biotechnology, Institute of Hydrobiology, Chinese Academy of Sciences, Wuhan 430072, China; liyi_0831@126.com (Y.L.); yeding@ihb.ac.cn (D.Y.); yhsun@ihb.ac.cn (Y.S.)
3 Laboratory for Marine Biology and Biotechnology, Qingdao National Laboratory for Marine Science and Technology, Qingdao 266237, China
4 Key Laboratory for Algal Biology, Institute of Hydrobiology, Chinese Academy of Sciences, Wuhan 430072, China
5 College of Life Sciences, University of Chinese Academy of Sciences, Beijing 100049, China
6 College of Advanced Agricultural Sciences, University of Chinese Academy of Sciences, The Innovative Academy of Seed Design, Chinese Academy of Sciences, Beijing 100049, China
7 Beijing Key Laboratory of Algae Biomass, Microalgae Biotechnology Center, SDIC Biotech Investment Co., LTD., State Development & Investment Corp., Beijing 100142, China
* Correspondence: huqiang@ihb.ac.cn (Q.H.); danxianghan@ihb.ac.cn (D.H.)

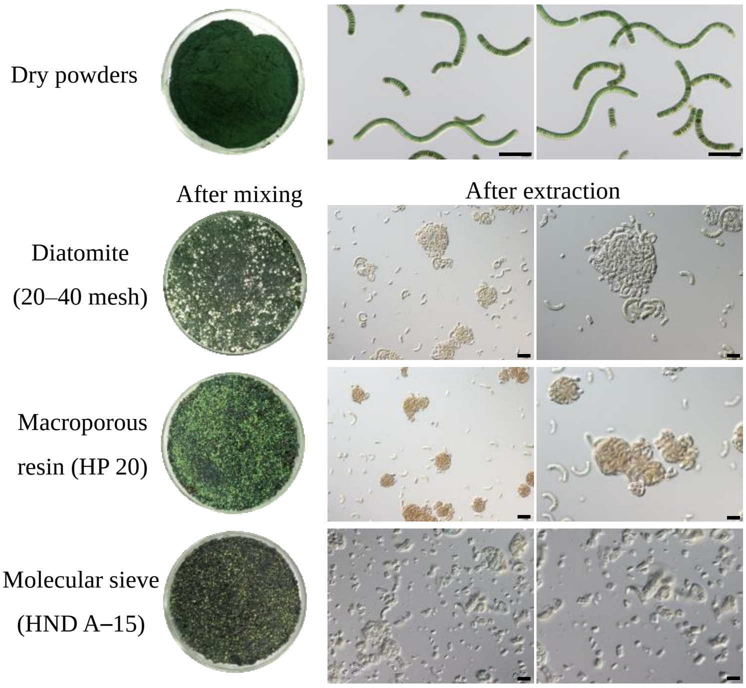

Figure S1. Comparison of the states of mixing porous materials with algal powders and the microscopic images of residues (Scale bar 50 μm).

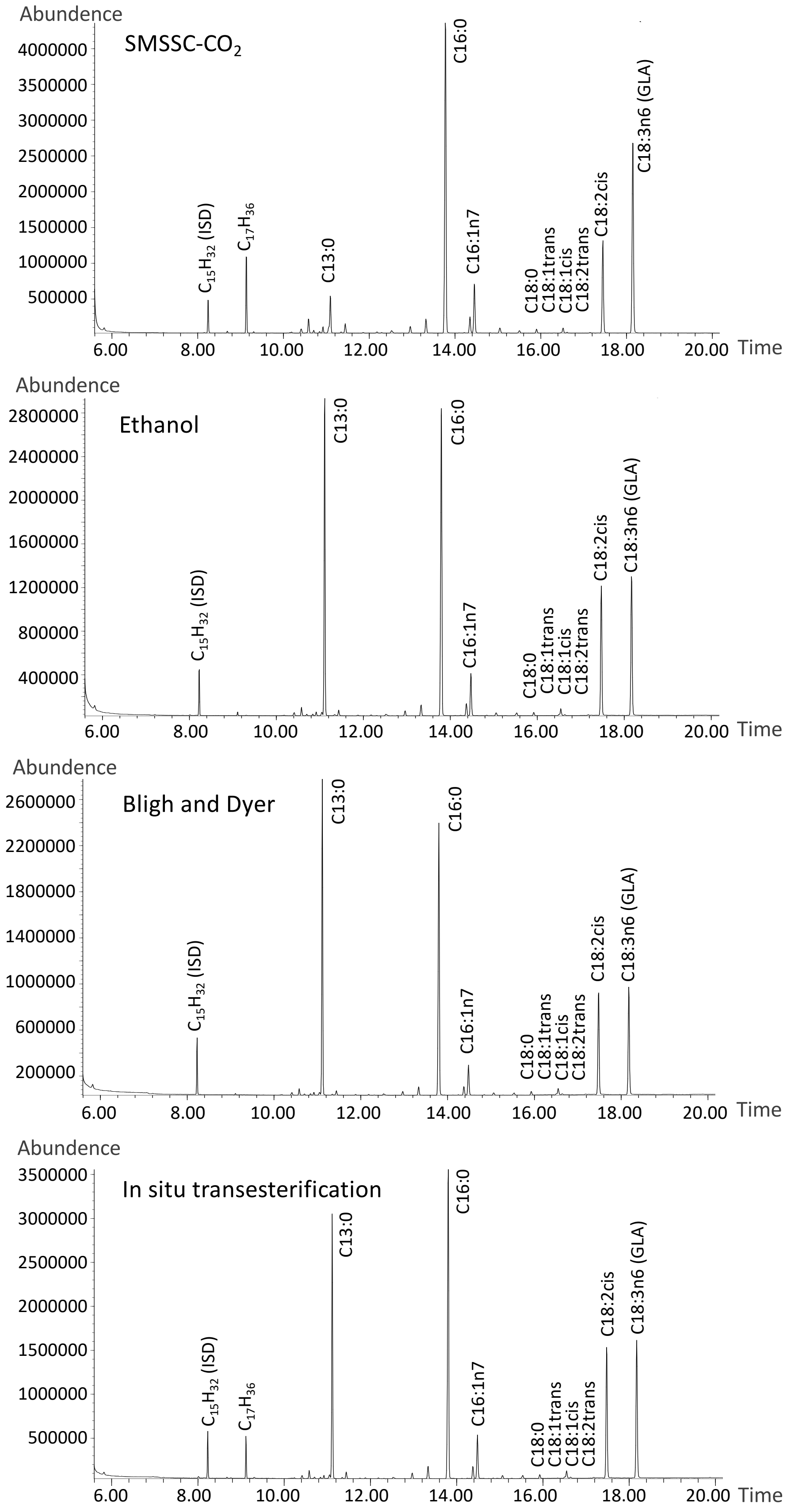

Figure S2. The chromatograms of fatty acid profiles obtained from the solid matrix-supported supercritical CO_2 (SMSSC-CO_2), ethanol, Bligh and Dyer, and in situ transesterification methods.
